# Short cervical lengths initially detected in mid-trimester and early in the third trimester in asymptomatic twin gestations: Association with histologic chorioamnionitis and preterm birth

**DOI:** 10.1371/journal.pone.0175455

**Published:** 2017-04-11

**Authors:** Jeong Woo Park, Kyo Hoon Park, Eun Young Jung, Soo-Hyun Cho, Ji Ae Jang, Ha-Na Yoo

**Affiliations:** 1 Department of Obstetrics and Gynecology, Inje University College of Medicine, Ilsan-Paik Hospital, Gyeonggi, Republic of Korea; 2 Department of Obstetrics and Gynecology, Seoul National University College of Medicine, Seoul, Republic of Korea; 3 Department of Obstetrics and Gynecology, Seoul National University College of Medicine, Seoul National University Bundang Hospital, Seongnam, Republic of Korea; University of Illinois at Urbana-Champaign, UNITED STATES

## Abstract

**Objective:**

To determine whether short cervical lengths (≤20 mm) that were initially detected in mid-trimester and early in the third trimester are independently associated with increased risks of subsequent histologic chorioamnionitis and spontaneous preterm birth (SPTB, defined as a delivery before 34 weeks) in asymptomatic women with twin pregnancies.

**Material and methods:**

This is a prospective study including 292 consecutive asymptomatic women with twin gestations. Cervical length measurements were carried out at 20 to 24 weeks’ gestation and at 28 to 32 weeks’ gestation. Both placentas of each twin pair were examined histologically after delivery. The generalized estimation equations models and logistic regression analysis were used for statistical analyses.

**Results:**

Multivariable generalized estimation equations analysis revealed that short cervical length at mid-trimester was independently associated with an increased risk for subsequent histologic chorioamnionitis, whereas short cervical length initially detected early in the third trimester was not. By using the likelihood of SPTB as an outcome variable, multivariable logistic regression analysis indicated that short mid-trimester cervical length and histologic chorioamnionitis were independently associated with a greater risk for SPTB. Similarly, based on the multivariable analysis, a short third trimester cervical length was independently and significantly associated with a greater risk for SPTB.

**Conclusions:**

In asymptomatic women with twin pregnancies, a short mid-trimester cervical length is independently associated with an increased risk of both subsequent histologic chorioamnionitis and SPTB, whereas a short cervical length initially detected early in the third trimester is independently associated with preterm delivery, but not subsequent histologic chorioamnionitis.

## Introduction

Twin pregnancies have increased during the last decade, reaching 33.2 per 1000 births in 2009, and have a six to ten times increased risk of preterm birth compared with singleton pregnancies [1]. Although the causes of preterm birth are multiple, in the context of twin pregnancies, uterine overdistention and intra-uterine infection/inflammation are traditionally recognized as major potential mechanisms of preterm birth [2, 3]. In the clinical perspective, ultrasonographic assessment of cervical length can be utilized as an effective tool to simultaneously reflect these two mechanisms of preterm delivery [4–7].

An association between shortened cervical length at 20 to 24 weeks’ gestation and preterm delivery has been established in asymptomatic women with twin pregnancies [8, 9]. A short cervical length at mid-trimester is also associated with an increased risk of intra-uterine infection/inflammation [5–7, 10]. However, it is unclear if the increased risk of preterm delivery in asymptomatic twins associated with short mid-trimester cervical length is related to the presence of intra-uterine infection/inflammation, as intra-uterine infection/inflammation also carries a higher risk of preterm delivery [10, 11]. On the other hand, several studies on asymptomatic twin pregnancies have highlighted that the relationship between a short cervical length and the risk of preterm delivery is affected by the gestational age at which the cervical length is measured; the lower the gestational age at the diagnosis of a short cervix, the higher the risk of preterm birth [12, 13]. In contrast to a short cervical length at mid-trimester, the effect of a short cervical length initially detected early in the third trimester on intra-uterine infection/inflammation and preterm delivery is unknown, despite the fact that cervical shortening in the third trimester is a relatively common finding in twin gestations [14]. Indeed, short cervical length initially detected early in the third trimester is generally considered as a normal physiologic change during pregnancy that does not have clinical implications, although evidence is lacking. The purpose of the study was to determine whether short cervical lengths that were initially detected in the mid-trimester and early in the third trimester are independently associated with increased risks of subsequent histologic chorioamnionitis and spontaneous preterm birth (SPTB) in asymptomatic women with twin pregnancies.

## Materials and methods

This study was a single-center prospective cohort study conducted at Seoul National University Bundang Hospital (Seongnamsi, Korea) between June 2008 and January 2015. The institutional review board (IRB) of the Seoul National University Bundang Hospital approved this study (project No. B-0804/056-001) and written informed consent was obtained from all study subjects. Women with twin pregnancies who attended routine antenatal clinics to undergo anomaly scan and cervical length measurements were consecutively recruited in the study at 20 to 24 weeks of gestation. Women were excluded for the following conditions: singleton and triplet or higher-order multiple pregnancy; prior or subsequent cervical cerclage; losses to follow-up after mid-trimester cervical length measurements; symptomatic preterm labor; preterm premature rupture of membranes; major congenital anomalies; and dead fetus. The women underwent an initial cervical length measurement at the time of routine ultrasound examination between 20 and 24 weeks’ gestation, followed 4 weeks later until 28 weeks, and then every 2 weeks until 32 weeks. Except for the women who delivered before 28 weeks’ gestation, cervical length measurements were carried out during each of the following time periods: 20 to 24 weeks’ gestation and 28 to 32 weeks’ gestation. The primary outcome measures were histologic chorioamnionitis and SPTB at < 34 weeks’ gestation. Additionally, we analyzed the data for SPTB at < 32 weeks’ gestation.

Transvaginal ultrasonography to measure cervical length was performed by Maternal Fetal Medicine faculties or fellows by using either a Voluson 730 Expert (GE Healthcare, Milwaukee, WI, USA) or an Aloka SSD 5500 (Aloka Co. Ltd., Tokyo, Japan) ultrasound machine equipped with a 6.0-MHz transducer. The detailed description of cervical length measurements was published elsewhere [15]. Cervical length was measured by placing the electronic markers at the furthest points between the internal os and external os, measuring it as a straight line. The shortest of three measurements obtained was taken as the cervical length. The women and their responsible obstetricians were not blinded to cervical length measurements and any interventions were instituted at the obstetricians’ discretion.

Immediately after delivery, placentas were collected and labeled as Ⅰ (1 cord clamp) or Ⅱ (no cord clamp) according to birth order. The placental specimens were processed in the pathology department according to the protocol of the College of American Pathologists [16]. Both placentas of each twin pair were used in the histopathologic analysis. After the maternal and fetal surfaces were grossly examined, the placental plate was sectioned at 1cm intervals and examined for any focal lesions. A full-thickness section of the placenta (including the maternal and fetal surfaces) was taken from the mid-zone of the placenta for further histological evaluation. A membrane roll was sampled, from the point of rupture to the edge of the placental disc, and a section of the umbilical cord (which was also sectioned at 1cm intervals) was submitted for histologic evaluation. Consequently, specimens for microscopic examination were taken for each case: one from an umbilical cord, one from a roll of fetal membranes, and one from full-thickness section of the placental disk parenchyma. Additional specimens were taken from any gross lesions. The presence of acute inflammation was noted and classified in each placenta as grade 1 or 2 according to criteria previously published [17]. Acute histologic chorioamnionitis was defined as the presence of acute inflammatory change in any tissue sample (amnion, chorion-decidua, umbilical cord, or chorionic plate). Funisitis was diagnosed by the presence of neutrophil infiltration into the umbilical vessel walls or Wharton's jelly. For each placenta, the total grade of histologic chorioamnionitis was calculated as the sum of histologic grades in the amnion (0–2), chorion-decidua (0–2), umbilical cord (0–2), and chorionic plate (0–2). Clinical chorioamnionitis was defined according to the criteria proposed by Gibbs et al. [18]. A short cervical length was defined as a cervical length of ≤ 20 mm. When multiple scans were performed on each subject, the shortest cervical length was used for defining a short cervical length in the study. SPTB was defined as delivery before 34 weeks’ gestation after spontaneous onset of preterm labor or premature rupture of membranes. The early third trimester was defined as 28 to 32 weeks of gestation.

Statistical analyses were performed using SPSS version 22.0 for Windows (IBM SPSS Statistics, Chicago, IL, USA). The Shapiro-Wilk test was used to assess whether data are normally distributed or not. Comparisons of continuous variables were performed with Student's *t* test or Mann-Whitney *U* test and proportions were compared with the *χ*^2^-test or Fisher’s exact test, as appropriate. For determining the associations of histologic chorioamnionitis and funisitis with the explanatory variable (i.e., short cervical length), we used generalized estimation equations (GEE) model to account for correlated binary responses from the same twin pair; thereafter, a multivariable GEE model was conducted to examine the relationship of histologic chorioamnionitis and funisitis to short cervical length after adjusting for baseline variables. On the other hand, the usual uncorrelated logistic regression analysis was performed to assess the association between pregnancy characteristics that did not produce cluster-correlated data (gestational age at measurement, short third trimester cervical length, and IVF) and SPTB. In these analyses wherein a single outcome is measured in the same subject, acute histologic chorioamnionitis and funisitis within a mother were entered as the presence of them in either or both twins, respectively. Only variables with *P* values of < 0.1 in the univariate analysis were entered in the multivariable analysis. All statistical analyses were performed by using a two-sided test with a significance level of 0.05.

## Results

During the study period, a total of 378 consecutive twin pregnant women were recruited at 20 to 24 weeks’ gestation for this study. Of these 378 women, 1 had a huge cervical myoma; 17 women underwent cervical cerclage because of a history of cervical incompetence or a short cervix; 61 women delivered outside of our hospital and were lost to follow-up, and 2 women had an incomplete data set. Five women with medically indicated preterm birth at < 34 weeks’ gestation were further excluded from the analysis (preeclampsia [n = 4] and twin-twin transfusion syndrome [n = 1]). Thus, 292 women were suitable for evaluating the relationships among a short mid-trimester cervical length, acute inflammatory lesions in the placentas and SPTB. The mean (SD) cervical length in mid-trimester was 36.1 (8.7) mm at a mean gestational age of 21.3 (1.2) weeks. The cervical length in mid-trimester was ≤ 20 mm in 12 women (4.1%). Histologic evidence of chorioamnionitis and funisitis was present in 14.0% (41/292) and 3.1% (9/292) of first-born twins and 11.0% (32/292) and 2.7% (8/292) of second-born twins, respectively. The prevalence of histologic chorioamnionitis and funisitis present in either or both twins was 17.5% (51/292) and 4.8% (14/292), respectively. The mean (SD) gestational ages at birth were 33.3 (5.4) weeks for women with histologic chorioamnionitis that was present in either or both twins and 36.2 (2.0) weeks for those in whom histologic chorioamnionitis was not present in either twin (*P* < 0.001).

Table 1 describes the clinical characteristics of the study population according to the presence or absence of short mid-trimester cervical length (≤ 20 mm). Women with short cervical lengths tended to have cervical lengths measured at a later gestational age, although they did not show statistical significance (*P* = 0.067). However, there were no differences in maternal age and rates of nulliparity, prior preterm births, chorionicity, and IVF. Women with short cervical lengths delivered significantly earlier and had a significantly higher median body mass index (BMI) at the time of mid-trimester ultrasound and higher risks of SPTB before 32 and 34 weeks' gestation than those with normal cervical length. Moreover, based on the univariate analyses, short cervical length at mid-trimester was significantly associated with the development of histologic chorioamnionitis, funisitis, total grade of histologic chorioamnionitis, and clinical chorioamnionitis (histologic chorioamnionitis, OR = 6.790, 95% CI = 2.185–21.096, *P* = 0.001; funisitis, OR = 8.405, 95% CI = 2.040–34.619, *P* = 0.003; total grade of histologic chorioamnionitis, OR = 15.739, 95% CI = 3.977–62.288, *P <* 0.001; GEE model in analyses). These associations remained significant after adjustment for potential confounders, such as gestational age and BMI at mid-trimester ultrasound (histologic chorioamnionitis, OR = 8.151, 95% CI = 2.568–26.869, *P* < 0.001; funisitis, OR = 6.303, 95% CI = 1.781–22.306, *P* = 0.004; total grade of histologic chorioamnionitis, OR = 628.967, 95% CI = 1.061–372888.866, *P =* 0.048; multivariate GEE model in analyses; clinical chorioamnionitis, OR = 31.109, 95% CI = 6.347–152.466, *P* < 0.001).

**Table 1:** Clinical characteristics and pregnancy outcomes of the study population according to the presence or absence of a short mid-trimester cervical length among 292 twin pregnancies.

	Mid-timester short cervix (≤ 20mm)		*P* value
	Absent (n = 280)	Present (n = 12)	
Maternal age (years)	32.8 ± 3.1	33.6 ± 5.4	.816
Nulliparity	77.1% (216/280)	75.0% (9/12)	.863
Prior preterm birth (< 37 weeks)	1.1% (3/280)	8.3% (1/12)	.155
Monochorionic	19.3% (54/280)	16.7% (2/12)	1.000
In vitro fertilization	53.6% (150/280)	75.0% (9/12)	.144
BMI at mid-trimester ultrasound (kg/m^2^)	23.9 ± 2.9	27.1 ± 5.3	.046
Gestational age at mid-trimester ultrasound (weeks)	21.3 ± 1.2	22.2 ± 1.7	.067
Discordant twin (weight discordancy ≥ 20%)	20.7% (58/280)	8.3% (1/12)	.296
Cesarean delivery	65.7% (184/280)	50.0% (6/12)	.264
Clinical chorioamnionitis	1.8% (5/280)	41.7% (5/12)	< .001
HCA in the first-born twin	12.5% (35/280)	50.0% (6/12)	< .001
A total grade of HCA in the first-born twin	0 (0–6)	1 (0–7)	< .001
Funisitis in the first-born twin	2.1% (6/280)	25.0% (3/12)	< .001
HCA in the second-born twin	9.7% (27/279)	41.7% (5/12)	.001
A total grade of HCA in the second-born twin	0 (0–7)	0 (0–6)	< .001
Funisitis in the second-born twin	2.5% (7/279)	8.3% (1/12)	.227
HCA in either or both twins	16.1% (45/280)	50% (6/12)	.002
Sum of total grade points of HCA in both twins	0 (0–13)	2 (0–13)	< .001
Funisitis in either or both twins	3.9% (11/280)	25% (3/12)	.001
SPTB < 32 weeks	5.4% (15/280)	58.3% (7/12)	< .001
SPTB < 34 weeks	8.4% (24/285)	75.0% (9/12)	< .001
Gestational age at delivery (weeks)	35.9 ± 2.5	28.6 ± 6.1	< .001

BMI, body mass index; SPTB, spontaneous preterm birth; HCA, histologic chorioamnionitis.

Data are given as the mean ± standard deviation, median (range) or % (n/N).

By using the likelihood of SPTB as the outcome variable, multivariable logistic regression analysis was performed to estimate the independent associations of a short mid-trimester cervical length and histologic chorioamnionitis with SPTB. Only four variables with *P* **<** 0.1 shown to be associated with SPTB in the bivariate analysis were included in the multiple logistic regression analysis: short mid-trimester cervical length, clinical chorioamnionitis, histologic chorioamnionitis, and funisitis. As shown in Table 2, short mid-trimester cervical length and the presence of histologic chorioamnionitis were independently and significantly associated with a greater risk for SPTB at < 32 weeks and < 34 weeks.

**Table 2:** Relationship of short mid-trimester cervical length and histologic chorioamnionitis with the risk of spontaneous preterm birth among 292 twin pregnancies, analyzed by multivariable logistic regression.

Variables		Odds ratio	95% confidence interval	*P* value
**1) Delivery within 34 weeks of gestation**				
Short mid-trimester cervical length (≤ 20mm)		18.223	3.794–87.538	< .001
Clinical chorioamnionitis		7.250	0.874–60.113	.066
HCA				.002
	HCA in neither twin	Reference		
	HCA in either twin	1.162	0.256–5.264	.846
	HCA in both twins	9.244	2.610–32.735	.001
Funisitis				.559
	Funisitis in neither twin	Reference		
	Funisitis in either twin	1.345	0.195–9.272	.763
	Funisitis in both twins	0.101	0.001–10.081	.329
**2) Delivery within 32 weeks of gestation**				
Short mid-trimester cervical length (≤ 20mm)		12.141	2.318–63.593	.003
Clinical chorioamnionitis		4.108	0.576–29.309	.159
HCA				< .001
	HCA in neither twin	Reference		
	HCA in either twin	2.651	0.526–13.363	.237
	HCA in both twins	16.583	4.156–66.160	< .001
Funisitis				.479
	Funisitis in neither twin	Reference		
	Funisitis in either twin	2.113	0.329–13.575	.431
	Funisitis in both twins	0.215	0.005–10.063	.434

HCA, histologic chorioamnionitis.

To analyze the relationship between short cervical length (≤ 20 mm) that was first detected in the early third trimester, acute inflammatory lesions in the placentas and SPTB, we further excluded 23 women [12 women due to short cervical lengths (≤ 20 mm) first detected in mid-trimester, 9 women due to preterm delivery at ≤ 28 weeks, 2 women due to missed follow-up scans]. Thus, a total of 269 women were suitable for this evaluation. The prevalence of a cervical length of ≤ 20 mm that was first detected in the early third trimester was 28.6% (77/269).

The clinical characteristics of the study population stratified according to the presence or absence of short cervical lengths (≤ 20 mm) first detected in the third trimester are shown in Table 3. There were no differences in demographic and clinical characteristics, except for a lower rate of cesarean delivery in the short third trimester cervical length group (57% vs. 70%, *P* = 0.047). Compared to women with normal cervical lengths, women with short cervical lengths in the third trimester delivered significantly earlier and had higher risks of SPTB before 32 and 34 weeks' gestation. However, a short cervical length first detected in the third trimester was not associated with subsequent development of histologic chorioamnionitis, funisitis or total grade of histologic chorioamnionitis (histologic chorioamnionitis, OR = 1.371, 95% CI = 0.647–2.904, *P* = 0.410; funisitis, OR = 1.074, 95% CI = 0.197–5.862, *P* = 0.934; total grade of histologic chorioamnionitis, OR = 1.371, 95% CI = 0.651–2.887, *P =* 0.406; GEE model in analyses). Also, a short cervical length first detected in the third trimester did not have an association with the subsequent risk of clinical chorioamnionitis.

**Table 3:** Clinical characteristics and pregnancy outcomes of the study population according to the presence or absence of short cervical length first detected in the early third trimester among 269 twin pregnancies.

	Short cervix in the third trimester (≤ 20mm)		*P* value
	Absent (n = 192)	Present (n = 77)	
Maternal age (years)	33.1 ± 3.1	32.3 ± 3.2	.080
Nulliparity	75.5% (145/192)	77.9% (60/77)	.676
Prior preterm birth (< 37 weeks)	1.0% (2/192)	1.3% (1/77)	1.000
Monochorionic	17.2% (33/192)	27.3% (21/77)	.062
In vitro fertilization	56.3% (108/192)	46.8% (36/77)	.158
BMI at the third trimester ultrasound (kg/m^2^)	26.0 ± 2.7	25.9 ± 3.2	.602
Gestational age at the third trimester ultrasound (weeks)	31.3 ± 1.0	31.3 ± 1.0	.969
Cervical length at the third trimester ultrasound (mm)	29.5 ± 5.9	14.2 ± 4.7	< .001
Cervical length at mid-trimester ultrasound (mm)	39.3 ± 6.3	32.4 ± 6.4	< .001
Discordant twin (weight discordancy ≥ 20%)	21.9% (42/192)	18.2% (14/77)	.500
Cesarean delivery	69.8% (134/192)	57.1% (44/77)	.047
Clinical chorioamnionitis	0.5% (1/192)	1.3% (1/77)	.491
HCA in the first-born twin	9.4% (18/192)	13.0% (10/77)	.381
A total grade of HCA in the first-born twin	0 (0–6)	0 (0–3)	0.402
Funisitis in the first-born twin	1.6% (3/192)	1.3% (1/77)	1.000
HCA in the second-born twin	7.3% (14/192)	9.2% (7/76)	.598
A total grade of HCA in the second-born twin	0 (0–4)	0 (0–3)	0.749
Funisitis in the first-born twin	2.1% (4/192)	2.6% (2/76)	.678
HCA in either or both twins	13.5% (26/192)	15.6% (12/77)	.664
Sum of total grade points of HCA in both twins	0 (0–8)	0 (0–6)	0.710
Funisitis in either or both twins	3.1% (6/192)	2.6% (2/77)	1.000
SPTB < 32 weeks	0.5% (1/192)	5.2% (4/77)	.025
SPTB < 34 weeks	2.1% (4/192)	13.0% (10/77)	< .001
Gestational age at delivery (weeks)	36.6 ± 1.2	35.8 ± 2.0	.015

BMI, body mass index; SPTB, spontaneous preterm birth; HCA, histologic chorioamnionitis.

Data are given as the mean ± standard deviation, median (range) or % (n/N).

Multivariable logistic regression analysis results, including covariates with *P* **<** 0.1 shown to be associated with SPTB in the bivariate analysis, are shown in Table 4. Short cervical length first detected in the third trimester was significantly associated with a greater risk for SPTB at < 34 weeks after adjusting for potential confounders (i.e., gestational age at the third trimester ultrasound, BMI at the third trimester ultrasound, and clinical chorioamnionitis).

**Table 4:** Relationship of various independent variables with the risk of spontaneous preterm birth among 269 twin pregnancies, analyzed by multivariable logistic regression.

Variables	Odds ratio	95% confidence interval	P value
**1) Delivery within 34 weeks of gestation**			
Gestational age at the third trimester ultrasound (weeks)	0.556	0.347–0.889	.014
BMI at the third trimester ultrasound (kg/m^2^)	1.172	0.984–1.396	.076
Short cervical length in the third trimester (≤ 20mm)	8.605	2.387–31.021	.001
Clinical chorioamnionitis	14.697	0.772–279.695	.074
**2) Delivery within 32 weeks of gestation**			
Gestational age at the third trimester ultrasound (weeks)	0.269	0.100–0.726	.009
Short cervical length in the third trimester (≤ 20mm)	13.292	0.749–235.898	.078
In vitro fertilization	0.000	0.000	.995
BMI at the third trimester ultrasound (kg/m^2^)	1.359	0.959–1.926	.085

BMI, body mass index.

Table 5 presents diagnostic indices of short cervical lengths initially detected in the mid-trimester and early in the third trimester to predict SPTB <34 weeks’ gestation, histologic chorioamnionitis, and funisitis. A short mid-trimester cervical length for these outcome variables showed high specificity and negative predictive value but were not sensitive with a low positive predictive value.

**Table 5:** Diagnostic indices in the prediction of spontaneous preterm delivery <34 weeks’ gestation, histologic chorioamnionitis, and funisitis.

Outcome	Sensitivity	Specificity	PPV	NPV
**SPTB at < 34 weeks**				
Mid-timester short cervix (≤ 20mm)	27.3 (9/33)	98.8 (256/259)	75.0 (9/12)	91.4 (256/280)
Short cervix in the third trimester (≤ 20mm)	71.4 (10/14)	73.7 (188/255)	13.0 (10/77)	97.9 (188/192)
**HCA in either or both twins**				
Mid-timester short cervix (≤ 20mm)	11.8 (6/51)	97.5 (235/241)	50.0 (6/12)	83.9 (235/280)
Short cervix in the third trimester (≤ 20mm)	31.6 (12/38)	71.9 (166/231)	15.6 (12/77)	86.5 (166/192)
**Funisitis in either or both twins**				
Mid-timester short cervix (≤ 20mm)	21.4 (3/14)	96.8 (269/278)	25.0 (3/12)	96.1 (269/280)
Short cervix in the third trimester (≤ 20mm)	25.0 (2/8)	71.3 (186/261)	2.6 (2/77)	96.9 (186/192)

PPV, positive predictive value; NPV, negative predictive value; SPTB, spontaneous preterm birth; HCA, histologic chorioamnionitis.

Data are given as % (n/N).

## Discussion

The principal findings of this study are as follows: (1) in asymptomatic women with twin pregnancies, a short mid-trimester cervical length (≤ 20 mm) is independently associated with an increased risk of subsequent histologic chorioamnionitis and is strongly associated with SPTB, independent of the presence of histologic chorioamnionitis; (2) a short cervical length initially detected early in the third trimester is associated with SPTB, but not subsequent histologic chorioamnionitis. These findings suggest that the role of short cervical lengths related to the risk of intra-uterine infection/inflammation may vary according to the age of gestation at the time of diagnosis, and that cervical length assessment early in the third trimester, as well as at mid-trimester, is useful to identify women at highest risk for SPTB.

This is the first study to examine the relationship between short cervical lengths according to trimesters of pregnancy, chorioamnionitis, and SPTB [Searches of PubMed (January 1966 through July 2016), EMBASE (January 1966 through July 2016), and The Cochrane Library were conducted using the following search terms: ‘histologic chorioamnionitis’, ‘preterm birth’, ‘twins’, and ‘short cervix’ or ‘short cervical length’]. We found that in asymptomatic women with twin pregnancies, women with short mid-trimester cervical lengths were more likely to have subsequent histologic chorioamnionitis than those with normal cervical lengths. This observation is in line with results of previous studies on multiple pregnancies by Guzman et al. and Pelaez et al. [10, 19] and suggests that the association between a short mid-trimester cervical length and the risk of SPTB may be potentially mediated by the present or subsequent development of intra-uterine infection/inflammation. Similarly, with respect to intra-amniotic inflammation, a significant correlation between the degree of cervical shortening and amniotic fluid cytokine levels was previously noted in women with singleton pregnancies and short cervical lengths in the mid-trimester [6]. Indeed, these findings are not unexpected, because a short cervical length may predispose to ascending infection of vaginal microbial flora, leading to intra-amniotic infection/inflammation and acute inflammatory lesions of the placenta, or vice versa. However, contrary to a short cervical length at mid-trimester, we found a lack of association between a short cervical length first detected early in the third trimester and subsequent histologic chorioamnionitis. This discrepancy is not clearly explained, but may be due to the difference in immunity to ascending infection from the lower genital tract that varies according to gestational age; the higher the gestational age, the higher the immunity to the microorganisms in the lower genital tract and the lower the risk of infection (fetoplacental, intra-amniotic, and neonatal infection) [2, 17, 20]. In support of our view regarding immune cell populations in the decidua under normal condition, Bartmann et al. recently reported that the amount of monocytes, immature dendritic cells, and T cells continuously increased during the course of pregnancy [21].

In line with previously published data [8, 9, 22, 23], a significant association of a short mid-trimester cervical length with SPTB was observed in the current study. We also found a significant association between short cervical length first detected early in the third trimester with a greater risk for SPTB, which is similar to the findings of Goldenberg et al. and Vayssiere et al. [22, 23]. They demonstrated that cervical length data obtained between 26 and 28 weeks correlate with the risk of preterm delivery in twin pregnancies [22, 23]. However, in terms of study design, our study is different from both studies [22, 23] in that we excluded women with short cervical lengths (≤ 20 mm) at mid-trimester, in order to evaluate the direct effect of a shortened cervical length first detected in the third trimester on the chorioamnionitis and SPTB. To date, no effective interventions have been shown to reduce the risk of SPTB in mothers of twins with a short cervix [24]; thus, implementation of routine cervical length screening may be limited in clinical practice. However, our data and those of other groups [22, 23] may provide evidence justifying serial cervical length measurements early in the third trimester in twin pregnancies, as this information can be used in antenatal preparation (i.e., antenatal corticosteroid administration and transfer of the mother to a tertiary facility) of targeted pregnancies identified as high risk for SPTB. Further studies are needed to investigate if the subset of women in whom a shortened cervical length is first identified in the early third trimester may also be appropriate candidates for the potential benefits of the interventions (i.e., cervical pessary, progesterone supplementation, and bed rest) that have limited evidence for their effectiveness in twins with a mid-trimester short cervix because mechanisms for preterm delivery arising from a short cervix could be different between the second and third trimester.

Our finding that a short mid-trimester cervical length in asymptomatic women with twin pregnancies is independently associated with a high likelihood of subsequent clinical chorioamnionitis and funisitis is in accordance with the results of previous studies on singleton gestations [25, 26]. They demonstrated a significant link between a short mid-trimester cervical length, and clinical chorioamnionitis, early onset sepsis and neonatal morbidity and mortality [25, 26]. Collectively, these findings suggest that a short mid-trimester cervical length may play an important role in the development of both fetal and maternal inflammatory responses, potentially through ascending infection [27, 28].

Our finding that higher BMI at mid-trimester ultrasound was associated with short mid-trimester cervical length is consistent with the results of previous studies on singleton gestations [29, 30]. Although we cannot fully explain these findings, they are related to the fact that higher BMI may induce uterine contractions and cervical ripening, leading to shortening of the cervix, by increasing factors involved in obesity, such as chronic low-grade inflammation and metabolic and hormonal alterations. In fact, several previous studies have reported the significant association between higher maternal BMI and SPTB [31, 32].

The current study has several limitations. First, the results of cervical length measurements were reported to the women and their managing obstetricians, which may have potentially led to the initiation of various clinical practices to reduce preterm birth, although no intervention (i.e. progesterone, cerclage, and bed rest) has been shown to be beneficial in women with twin pregnancies and a short cervix at mid-trimester [24, 33, 34]. Second, our data were analyzed at a cut-off cervical length of 20 mm to define short cervical length, which may affect outcomes. Although the cut-off values to define a short cervical length were previously reported to be 20 to 35 mm in twin gestations, a recent meta-analysis has suggested 20 mm as the best cut-off for the prediction of SPTB at < 32 and 34 weeks’ gestation [8]. Third, the analysis in the current study was limited by the small number of cases (n = 12) of a short mid-trimester cervical length. The main strengths of our study are 1) the prospective nature of data collection; 2) the relatively large sample size; 3) 100% of placentas were examined, distinguishing the placenta of the first baby from that of the second baby; 4) application of the GEE approach for correlated binary data to avoid making misleading conclusions; and 5) the serial measurements of cervical lengths by transvaginal ultrasound from second trimester to third trimester.

## Conclusions

Our study has shown that in asymptomatic women with twin pregnancies, a short mid-trimester cervical length is independently associated with an increased risk of both subsequent histologic chorioamnionitis and preterm delivery, whereas a short cervical length initially detected early in the third trimester is independently associated with preterm delivery, but not subsequent histologic chorioamnionitis.

## Supporting information

S1 File Raw data. (SAV)

S2 File GEE model. (SAV)
